# The role of the WatchPAT device in the diagnosis and management of obstructive sleep apnea

**DOI:** 10.3389/frsle.2023.1148316

**Published:** 2023-08-16

**Authors:** Christina D. Campbell, Imran Sulaiman

**Affiliations:** ^1^Department of Respiratory Medicine, Royal College of Surgeons in Ireland, Dublin, Ireland; ^2^Department of Respiratory Medicine, Beaumont Hospital, Beaumont, Dublin, Ireland

**Keywords:** obstructive sleep apnea, WatchPAT, telemedicine, sleep disorders, central sleep apnea (CSA)

## Abstract

Obstructive sleep apnea (OSA) is a common condition affecting an estimated 936 million individuals worldwide, leading to a considerable demand for diagnostic services. Polysomnography, the current gold standard for diagnosis, is resource intensive and inconvenient for patients and healthcare providers. The WatchPAT is an unobtrusive device for home OSA diagnosis. It utilizes peripheral arterial tomography in conjunction with heart rate, oximetry, actingraphy, and respiratory movements for the diagnosis of OSA. It has good correlation with polysomnography for OSA diagnosis and also reports sleep time and sleep staging. The WatchPAT device has reported sensitivities of 81–95%, specificities of 66–100%, positive predictive values of 79–96%, and negative predictive values of 92% for the determination of the apnea–hypopnea index (AHI). It has also been studied and its use validated in a variety of patient populations, including children, older adults, pregnant women, and those with comorbid medical conditions. The device has also been adopted for use in screening for cardiac arrhythmia and central sleep apnea, although neither use has become widespread. With the emergence of telemedicine and an increasing demand for sleep services, the WatchPAT device can be a useful aid in OSA diagnostics.

## Introduction

Obstructive sleep apnea (OSA) affects an estimated 936 million individuals worldwide, of whom approximately 425 million individuals require treatment (Benjafield et al., 2019), leading to a considerable demand for OSA diagnostics. The current gold standard for diagnosis, polysomnography, has considerable drawbacks and is resource intensive. As a consequence, newer less invasive diagnostic modalities have emerged. One such modality is the WatchPAT (WP) device. The aim of this review is to discuss the WP device, its role in OSA diagnosis in various patient populations, and other uses for the device that have emerged.

### Sources and search strategy

References used for this review were identified from PubMed and Medline searches. Search terms used included “WatchPAT,” “Peripheral Arterial Tomography,” and “PAT” in conjunction with specific disorders and other investigations, including “polysomnography (PSG),” “obstructive sleep apnoea,” “sleep apnoea,” “central sleep apnea,” and “sleep staging.”

## Background

OSA is caused by recurrent episodes of upper airway collapse during sleep, which results in a reduction (hypopnea) or absence (apnea) of airflow, oxygen desaturation, and arousal. Arousals contribute to symptoms including sleep fragmentation, unrefreshing sleep, and excessive daytime sleepiness. It is then graded in severity on the apnea-hypopnea index (AHI) according to the average number of hypopneas and apneas during sleep. OSA is associated with increased rates of road traffic accidents (Terán-Santos et al., 1999), workplace accidents (Garbarino et al., 2016), cardiovascular disease (Peppard et al., 2000; Punjabi et al., 2009), and diabetes (Wang et al., 2022), among others.

Polysomnography (PSG) is the current gold standard for OSA diagnosis. It measures sleep time, stage, and arousals using an electroencephalogram, airflow using a thermistor, respiratory efforts with chest and abdominal bands, oxygen saturation, body position, leg movements, and snoring, with or without video monitoring. PSG can be performed with direct monitoring in a sleep laboratory (Type 1 study) or unattended at home or in the laboratory (Type 2 studies). However, polysomnography has significant drawbacks. It is labor intensive, intrusive for patients, and expensive for the health care provider.

Given the limitations of inpatient PSG, home sleep apnea testing (HSAT) has become commonplace and has been further accelerated by the COVID-19 pandemic. HSAT is broadly considered under two clinical pathways: the multiple access outpatient pathway and the telemedicine pathway. Under the multiple access pathway, the patient attends an outpatient appointment, then returns to pick up their HSAT device, returns the device, and attends a final appointment for results. The telemedicine pathway involves telemedicine consultations and direct receipt of the testing device by the patient at home. Home sleep apnea tests (HSATs) have proved popular and may be either Type 3 studies, which measure at least two respiratory variables (respiratory effort, flow), oxygenation saturation, and a cardiac variable, or Type 4 studies, which measure only one or two parameters, generally oxygen saturation and heart rate. The American Academy of Sleep Medicine (AASM) guidelines state that an HSAT should incorporate at least the following: nasal pressure, chest and abdominal movements, oximetry or peripheral arterial tomography with oximetry and actingraphy (Kapur et al., 2017).

## WatchPAT technology

The WatchPAT device uses peripheral arterial tonometry (PAT). PAT technology is based on the variations in peripheral vascular resistance during sleep and arousals due to fluctuations in sympathetic nerve activity. During non-REM sleep, sympathetic activity is lower, with a consequent reduction in blood pressure and heart rate (Somers et al., 1993). During REM sleep, this activity increases above the levels observed during wakefulness, with blood pressure and heart rate increasing in tandem (Somers et al., 1993).

Arousals from sleep result in bursts of sympathetic activity, which in turn result in blood pressure surges and tachycardias (Schnall et al., 1999). These hemodynamic changes lead to increased peripheral vascular resistance. Our fingertips contain dense vascular beds with high contractions of sympathetic alpha receptors. Apnea- or REM sleep-induced activation of the sympathetic nervous system leads to the activation of these receptors, with resultant peripheral vasoconstriction, increased vascular tone, and decreased blood flow at the fingertip (Schnall et al., 1999). This can be detected by finger probe polysomnography. Studies have demonstrated a good correlation (r = 0.82) between PAT-detected apneas and those detected with EEG during PSG in both adult and pediatric populations (Pillar et al., 2002, 2003; O'Brien and Gozal, 2007).

**Figure 1:**
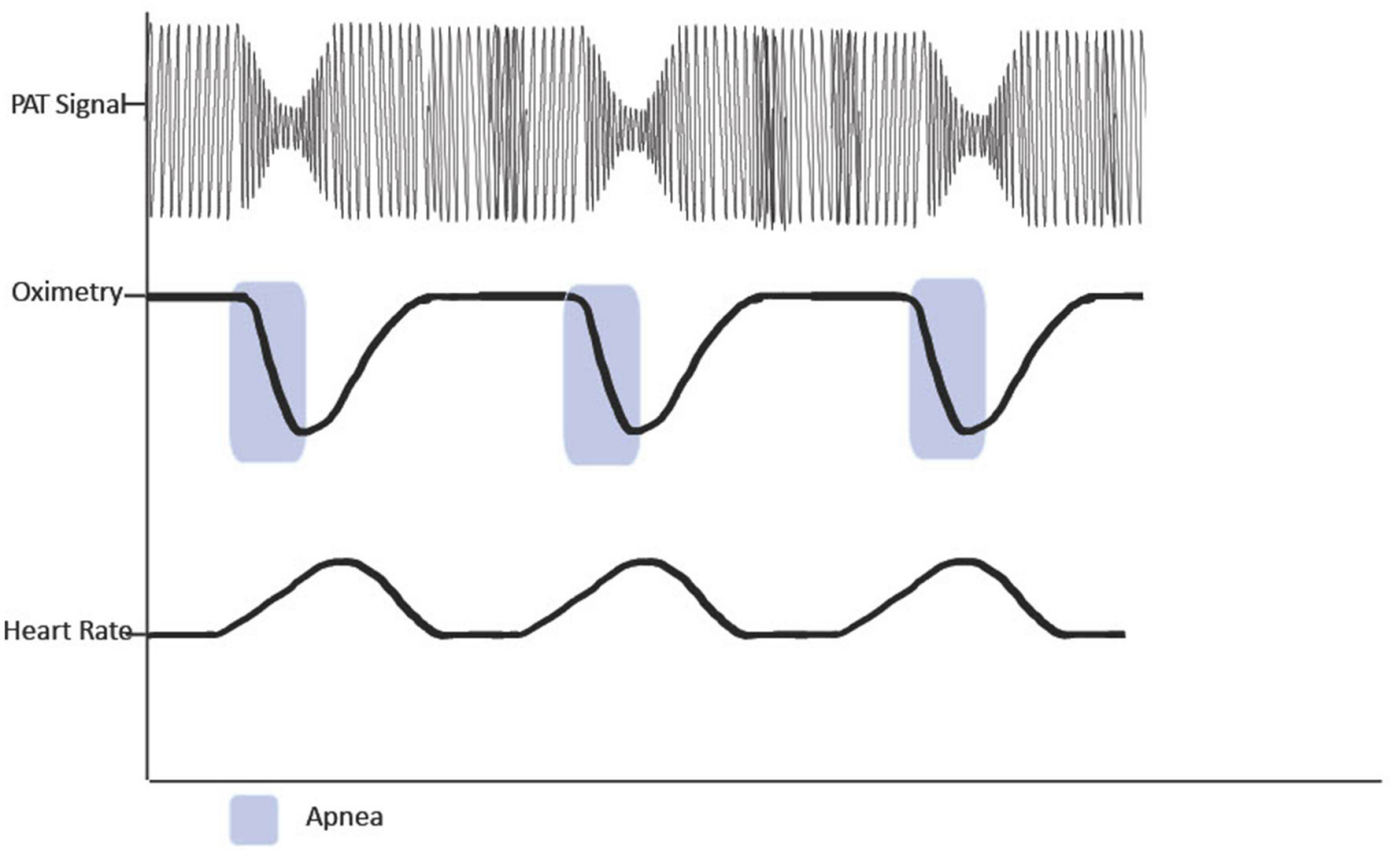
Figure demonstrating reduction in PAT signal following apnoic events.

Obstructive sleep apnea can be detected and diagnosed using PAT technology in conjunction with oximetry, heart rate, actingraphy, and respiratory movements (Penzel et al., 2004) (Figure 1). Additionally, WatchPAT technology allows for better assessment of sleep stages, including total sleep time, rather than total recording time, which is frequently used by HSAT (Hedner et al., 2011).

### The WatchPAT and sleep staging

The vast majority of home sleep studies do not record sleep itself, as they do not have access to EEG data. Instead, they rely on recording time. The WatchPAT device utilizes actingraphy (Hedner et al., 2004) and a PAT signal to distinguish between waking time, sleep, REM, and non-REM (NREM) sleep (Herscovici et al., 2007), of which the latter is further subdivided into light or deep sleep (Bresler et al., 2008). Traditionally, NREM sleep has been classified into four stages by PSG: stage 1 (N1), drowsiness; stage 2 (N2), light sleep; and finally stages 3 and 4 (N3 and N4), deep sleep. Instead, the WatchPAT divides NREM sleep into light sleep (stages 1 and 2) and deep sleep (stages 3 and 4).

The WatchPAT then combines PAT signal and actingraphy to develop an automatic sleep-stage detection algorithm, using the PAT software package (Medical, 2002b). However, it is important to appreciate that the evidence for WP sleep staging is weaker than that for the gold standard, PSG. In a large multicenter study, researchers compared PSG and WP sleep staging and found only moderate agreement on the sleep stage (Cohen κ coefficient = 0.475) (Hedner et al., 2011). A smaller study, designed to assess the efficacy of the WatchPAT-200 (WP-200) in the diagnosis of OSA, found low agreement between PSG and light sleep (ICC = 0.495, *p* < 0.001), very low agreement for REM sleep (ICC = 0.237, *p* = 0.044), and no agreement for deep sleep (*p* = 0.514) (Ceylan et al., 2012).

### Diagnosis of OSA by WatchPAT

There are currently three generations of the WatchPAT device: WatchPAT-100, 200, and 300 (WP-100, WP-200, and WP-300, respectively), as well as a disposable model, WatchPAT-ONE. All generations utilize PAT, oximetry, heart rate, and actingraphy, with various firmware and hardware upgrades in each generation. The most recent model, WP-300, contains an updated snoring, and body position sensor to monitor chest movements and diagnose central sleep apnea (Medical, 2020). Multiple studies have compared most devices with PSG, either by carrying out simultaneous PSG and WP studies or with studies on separate nights and in-lab and at-home studies. We have outlined in the following section the data relevant to OSA diagnosis for each generation of WP device in comparison to polysomnography for the diagnosis of obstructive sleep apnea.

At present, we know from several studies that the WP-100 has excellent correlation for AHI (Zou et al., 2006; Choi et al., 2010), respiratory desaturation index (RDI) (Bar et al., 2003; Penzel et al., 2004; Zou et al., 2006; Hedner et al., 2011), and oxygen desaturation index (ODI) (Penzel et al., 2004; Zou et al., 2006), with a reported sensitivity of 100%, a specificity of 83%, a positive predictive value (PPV) of 95%, and a negative predictive value (NPV) of 100% for the diagnosis of OSA compared to PSG (Choi et al., 2010).

Similarly, in a comparison with PSG, the WP-200 has been reported to have an excellent correlation for AHI (Pang et al., 2007; Ceylan et al., 2012; Onder et al., 2012; Weimin et al., 2013; Körkuyu et al., 2015; Gan et al., 2017; Tondo et al., 2021), RDI (Ceylan et al., 2012; Yuceege et al., 2013), ODI (Ceylan et al., 2012; Onder et al., 2012; Yuceege et al., 2013; Tondo et al., 2021), and mean oxygen saturations (Onder et al., 2012; Yuceege et al., 2013; Körkuyu et al., 2015). It also has a reported sensitivity of 81–95%, a specificity of 66–100%, a PPV of 79.4–96%, and an NPV of 92% for AHI (Ceylan et al., 2012; Weimin et al., 2013; Tondo et al., 2021). For RDI, a sensitivity of 89%, a specificity of 77%, a PPV of 82%, and an NPV of 86% are reported (Yuceege et al., 2013) (Table 1).

**Table 1:** Summary of studies comparing WatchPAT and polysomnography.

**References**	**WP device**	**Comparison device**	**Study population**	**AHI**	**RDI**	**ODI**	**Mean saturations**	**Sleep time**
Bar et al. (2003)	WatchPAT-100	Level 1 PSG	N = 102 (No gender breakdown)	-	R = 0.88, *p* < 0.001	-	-	-
Ceylan et al. (2012)	WatchPAT-200	Level 1 PSG	N = 51 (No gender breakdown)	AHI < 15: Sensitivity 0.93 Specificity 66 PPP 0.794 NPV 0.85 AHI < 30 Sensitivity 0.88 Specificity 0.8 PPV 0.71 NPV 0.92	ICC = 0.961, CI 0.858–0.951, *p < * 0.001	ICC = 0.877, CI = 0.794–0.928, *p < * 0.001	-	-
Choi et al. (2010)	WatchPAT 100	PSG	N = 25 (84% men)	R = 0.94, *p* < 0.001 Sensitivity 1 Specificity 0.83 PPV 0.95 NPV 1	-	-	-	-
Gan et al. (2017)	WP-200	PSG	N = 20 (90% men )	R = 0.94, *p < * 0.0001	-	-	-	R = 0.6228, *p < * 0.0034
Hedner et al. (2011)	WP-100	PSG	*n* = 228 (No gender breakdown.)	-	ICC = 0.87, *p < * 0.005	-	-	ICC = 0.79, *p < * 0.01
Körkuyu et al. (2015)	WP-200	Level 1 psg	*N* = 30 (83.8%)	R = 0.802, *p < * 0.001 PPV 0.96	-	-	Mean WP 93.1%, PSG 92.6%, *p < * 0.001	r = 0.246, *p =* 0.184
Onder et al. (2012)	WatchPAT-200	Level 1 PSG	N = 59 (64% men )	Group 1: r = 0.92 *p < * 0.001 Group 2: R = 0.94 *p < * 0.001	-	Group 1: R = 0.97 *p < * 0.001 Group 2:r = 0.99 *p < * 0.001	Group 1: R = 0.89, *p < * 0.001 Group 2: R = 0.96, *p < * 0.001	Group 1: R = 0.62, *p =* 0.003 Group 2: R = 0.24, *p =* 0.23
Pang et al. (2007)	Unspecified	Level 1 PSG	N = 37 (33%)	R = 0.9288, *p < * 0.0001 AHI > 5 Sensitivity 0.94 Specificity 0.8	-	-	-	R = 0.5815;, *p =* 0.005
Penzel et al. (2004)	WP-100	Level 1 PSG	N =21 (No gender breakdown.)	R = 0.89, *p < * 0.01	R = 0.89, *P < * 0.01	R = 0.87, *p < * 0.01	-	r = 0.15
Tondo et al. (2021)	WP-200	PSG	N = 47 (62% men )	R = 0.86, *p < * 0.001 Sensitivity 0.81, Specificity 0.73	-	0.93, *p < * 0.0001	-	-
Yuceege et al. (2013)	WP-200	Daytime PSG	N = 90 (100% men)	-	Sensitivity 0.89 Specificity 0.77 PPV 0.82 NPV 0.86	R = 0.923, *p < * 0.0001	R = 0.76, *p < * 0.0001	-
Zou et al. (2006)	WP-100	At home PSG	N = 106 (56% men)	R = 0.9, *p < * 0.0001	R = 0.88, *P < * 0.0001	R = 0.92, *p < * 0.0001	-	Mean difference 0.2 ± 1.1 h

The most recent WatchPAT device, WP-300, uses a finger probe for oximetry and polysomnography and a chest probe to record snoring, body position and respiratory effort. No data on the WP-300 have yet been published.

A cost-effectiveness study using an unspecified WP device found that the telemedicine pathway was more expensive for the healthcare provider but significantly cheaper for patients, indicating that a telemedicine service with WP devices would be acceptable to the patient population (Di Pumpo et al., 2022). Use of the WP device rather than full PSGs in traditional outpatient services has been shown to reduce time to OSA diagnosis and treatment, leading to overall cost savings (Phua et al., 2021).

### OSA treatment and WatchPAT technology

The WP has also been examined in the assessment of patients with OSA receiving continuous positive airway pressure (CPAP) therapy, which is the mainstay of treatment for individuals with OSA and excessive daytime sleepiness, or OSA and hypertension (Patil et al., 2019). One study compared simultaneous in-laboratory PSG and WP-100 assessment of patients on CPAP therapy, demonstrating good agreement between the WP and PSG for residual RDI (sensitivity of 86% and specificity 47% for RDI >5) (Pittman et al., 2006). An additional, non-inferiority study compared standard PSG diagnosis and titration of CPAP therapy with WP-100 in two separate groups. CPAP adherence and clinical outcomes were similar in both groups (Berry et al., 2008). Given the scarcity of PSG availability, many sleep centers use limited sleep studies (LSS) for the assessment of CPAP efficacy. One study compared LSS with WP analysis for residual AHI on CPAP; interestingly, the WP device detected a higher rate of residual Sleep Disordered Breathing (SDB) than the LSS (Schöbel et al., 2018).

## Limitations of WatchPAT in OSA

Although all these studies report high correlations between WP and PSG, with a proportion reporting high sensitivity and specificity, some have reported that the WP can underscore AHI at the mild range and overscore at the high range (Gan et al., 2017). A further study examined the correlation but also concordance for OSA diagnosis between the WP and PSG. Diagnostic accuracy was high in the moderate and severe OSA cohorts, with a sensitivity of 91%, a specificity of 61%, and negative and positive predictive values of 76 and 83% respectively. Conversely, of those assessed by the WP as having mild OSA, only 49.6% were deemed by PSG to have mild disease, with 20.4% having moderate or severe disease and 30.1% having no OSA (Ioachimescu et al., 2020). This suggests that further clarification is required in cases of high pre-test possibility or a negative test where symptoms are present. Additionally, in a meta-analysis of 17 studies comparing simultaneous PSG and WP, pooled specificities of 94, 92, and 74% and sensitivities of 44, 72, and 87% for AHI thresholds of 5, 15, and 30 events/h, respectively, were calculated (Iftikhar et al., 2022).

Importantly, in the vast majority of studies comparing WP and PSG, the majority of participants were men, with a mean 68% male majority in all studies, and some study populations containing 90–100% male participants (Yuceege et al., 2013; Gan et al., 2017). While this probably reflects the male preponderance in OSA, it does mean that the WP has been understudied in the female population; however, the results may not be generalizable.

It is also important to note that the WP device is not suitable for OSA diagnosis in all patients, and there are some contra-indications for its use. The manufacturer states that the device should not be used in adults taking short-acting nitrates or alpha blockers, those using a permanent pacemaker with atrial pacing and without sinus rhythm, or those with sustained non-sinus cardiac arrhythmia (Schnall et al., 2022).

Phentolamine, an alpha blocker, has been shown to induce peripheral vasoconstriction and decrease pulse wave amplitude during finger plethysmography, both in healthy controls (Grote et al., 2003) and in individuals with severe OSA (Zou et al., 2004). Although these studies were small, both strongly indicate that alpha blockers are likely to interfere with the diagnostic capabilities of a WatchPAT device. A specific study on the effect of nitrates on peripheral tomography in OSA has not yet been performed. Pharmacokinetic studies have demonstrated that both nitroglycerin and isosorbide dinitrate induce peripheral vasodilation and influence finger pulse plethysmography, which suggests that they would also interfere with the PAT signal (Schnelle et al., 1981; Bass et al., 1989). The manufacturers state that a wash-out period of 3 h may be adequate for doxazosin; however, this is based on a study where only five of the 106 participants used an alpha blocker.

The effect of cardiac pacing and cardiac arrhythmias other than atrial fibrillation on WatchPAT diagnosis has not been formally evaluated, but the manufacturers state that they presume that it would interfere with the WP algorithms that derive AHI, among others. Although this recommendation is not evidence based, it would be prudent to avoid the use of WP in these cases, if possible.

Additionally, given that WP technology relies on vascular tone, conditions such as arterial stiffness or atherosclerosis may interfere with its diagnostic capabilities. Brachial-ankle pulse wave velocity (baPWV) has been used as a surrogate measurement for arterial stiffness and is a known predictor of cardiovascular disease (Kim et al., 2014). A comparative study of WP and PSG measured baPWV and found that participants with an elevated baPWV have a low correlation between WP-AHI and PSG-AHI. Notably, in those with a pulse wave velocity that the investigators determined as high, there was no correlation between WP and PSG AHI (r = 0.4, *p* = 0.04) (Kinoshita et al., 2018). Although this was a small study of 61, predominantly male, patients with a high prevalence of cardiovascular co-morbidities, it is still a reasonable assumption that, in a population at high risk of cardiovascular disease and arterial stiffening, with a high pre-test probability of OSA, a negative WP study may require further PSG clarification.

The WatchPAT has been used to good effect for OSA diagnosis across a range of conditions and research studies, including asthma, diabetes, congestive cardiac failure, myasthenia gravis, and following surgical interventions for OSA (Park et al., 2014; Yeh et al., 2015; Shinoda et al., 2019; Carey et al., 2023). Despite some critical studies, it has proved to be a good tool that has been widely adopted for the diagnosis of OSA. In those with a negative WatchPAT study and significant symptoms, PSG clarification may be needed; however, this is often the case with other HSATs.

## OSA diagnosis in other patient populations

### Pediatrics

SDB affects 1–3% of all children, causing, among other effects, sleep disruption, daytime cognitive impairment, and behavioral problems (Evans et al., 2023). SDB encompasses upper airway obstruction in children with otherwise normal development as well as in those with other underlying conditions. Full PSG is the gold standard for diagnosis of SDB, but as with adults, it is burdensome and costly. Moreover, some children may find PSG difficult to tolerate.

The WatchPAT device is currently approved for use in children from 12 years of age weighing >65 kg in America, Europe, and Japan (Medical, 2002a). The WP-200 has been studied in children aged 8 to 15 years. Compared to simultaneous Level 1 PSG, the WP had an excellent agreement for AHI (ICC = 0.89) and ODI (ICC = 0.87). The device used the same algorithm as that used for adults, which may under-detect respiratory events in the pediatric population (Tanphaichitr et al., 2018). In another study specifically addressing the diagnosis of OSA in adolescents (ages 13–17 years), the WP-200 had a sensitivity of 100% and a specificity of 96% for an AHI > 5 events/h compared to PSG (Choi et al., 2018).

The WP has also been studied in children under the age of 12 with symptoms suggestive of pediatric SDB (PSDB) but negative nocturnal pulse oximetry. Nocturnal pulse oximetry is often used as a screening tool for PSDB, as it is widely available, inexpensive, and simple to carry out. A study examined the use of WP rather than PSG following a negative pulse oximetry reading. WP detected PSDB in 35.7% of children with an RDI of >5. An AHI criterion of >1 was fulfilled by 60.7% of children (Serra et al., 2017). Given that all the population had previously had a negative pulse oximetry reading, the WP may be a more useful screening tool in this population. There are some disadvantages to WP device use in children. A pediatric finger probe is not available, and children under the age of five or those will smaller fingers will not be able to use it.

### Pregnancy

SDB during pregnancy is associated with adverse outcomes, such as gestational hypertension, and is therefore important to detect. It is generally presumed to be related to gestational weight gain. PSG is inconvenient during pregnancy, so the WP would be a simple alternative. A study compared ambulatory PSG and WP-200 assessment of third-trimester pregnant women, some of whom were at high risk and some at low risk of SDB. Correlations between WP and PSG for total sleep time (r = 0.76, *p* < 0.001), AHI (r = 0.76, *p* < 0.001), RDI (r = 0.68, *p* < 0.001), and mean oxygen saturations (r =0.94, *p* < 0.001) were all high; however, only low correlation was found for sleep stages (r = 0.1–0.32 for each sleep stage) (O'Brien et al., 2012). Additionally, WP had a sensitivity of 88%, a specificity of 87%, a negative predictive value of 70%, and a positive predictive value of 96% for an AHI >5 events/h (O'Brien et al., 2012). This suggests that the WP could be a useful tool for detecting SDB in pregnancy.

### Patients with other medical conditions

The WatchPAT device has been examined in the diagnosis of OSA with a number of other conditions and co-morbidities.

For example, OSA is common in individuals with Down syndrome, with a reported prevalence of up to 78% (Giménez et al., 2018). A feasibility study into the utility of WatchPAT in a population of individuals with Down syndrome and signs and symptoms consistent with OSA found an OSA prevalence of 95%. Importantly, 69% of this study population found the WatchPAT device acceptable (Alma et al., 2022). Given that PSG is a burdensome investigation for any individual and those with intellectual disabilities often find some investigations difficult to tolerate, WatchPAT may provide a useful diagnostic tool in this population. As the study did not have a comparison group, the accuracy of the WatchPAT diagnosis in this population cannot be commented on.

For our growing elderly population, one study evaluated PSG and WatchPAT-200 in 56 individuals, comparing a younger age group (20–35 years) with an older group (50–65 years). Good agreement between PSG and WP was found for AHI and oxygen saturations in both groups, suggesting that WP can be reliably used in those populations. However, caution may still be needed for patients over 65 years old (Onder et al., 2012). As we age, vascular stiffness increases, and vascular compliance reduces. Moreover, cardiovascular co-morbidities are more prevalent in the elderly population. These factors could all influence the ability of the WatchPAT device to diagnose OSA in the elderly population.

Chronic obstructive pulmonary disease (COPD) is a common condition that often overlaps with OSA. Given that COPD can itself lead to reduced oxygen saturation, many studies evaluating WP have excluded individuals with COPD. However, two studies in which the majority of patients had moderate COPD have directly compared the PSG and WP-200 analysis of individuals with COPD. The investigators found good correlation in AHI (r = 0.85, *p* < 0.001) between PSG and WP, including in those with moderate to severe disease, demonstrating that the WP device can be used to diagnose OSA in this patient population (Holmedahl et al., 2019; Jen et al., 2020).

Sleep apnea is a recognized risk factor for cardiovascular disease, including atrial fibrillation (AF). OSA is common in individuals with AF, with an estimated prevalence ranging from 49 to 62% (Gami et al., 2004; Stevenson et al., 2008), and it increases the rate of AF after electrical cardioversion (Mazza et al., 2009). Moreover, treatment of OSA leads to a reduction in the risk of AF recurrence (Qureshi et al., 2015; Shukla et al., 2015). Generally, studies examining WP have excluded patients with cardiac arrhythmia, owing to the concern that the arrhythmia may interfere with PAT amplitude and rate changes. In a study examining the usefulness of WP for the diagnosis of OSA in patients with AF, the authors found that WP is a suitable diagnostic tool for OSA in this population. AF does not appear to interfere significantly with the PAT signal or the time used for analysis. WP had a sensitivity of 88% and a specificity of 63% for the diagnosis of OSA compared to PSG, with an overall agreement in sleep staging of 62%, similar to that found in the general population (Tauman et al., 2020). An additional study used the WatchPAT-ONE disposable device to screen for SDB in a population with AF using a telemedicine model. Here, a prevalence of 55% for moderate to severe SDB was found by the WP device, again indicating that the WP is a useful tool for SDB screening in individuals with AF (Verhaert et al., 2022).

## Other uses for the WatchPAT device

### Diagnosis of atrial fibrillation

From the above findings, it is clear that the WatchPAT device is suitable for diagnosis of OSA in individuals with AF. As AF is common in patients with SDB, a team attempted to find whether the WP device could detect cardiac arrhythmias during a sleep study. Using the WP-200 device, they performed simultaneous WP and PSG analysis in a population suspected of SDB with co-existing congestive cardiac failure or AF. The ECG was scored manually on the PSG and blinded to the WP analysis. It is known that the PAT signal directly relates to ventricular contraction and, hence, can be used to detect the irregularly irregular pattern of AF. The WatchPat used a novel automatic algorithm, based on the PAT signal, and was found to have moderate sensitivity (77%) but high specificity (99%) for the detection of AF lasting longer than 6 min (Pillar et al., 2022). The results indicate that the WP device may be a useful screening tool for AF in a population being evaluated for SDB and could flag patients who need further investigation.

### Diagnosis of central sleep apnea

Central sleep apnea (CSA) syndromes are characterized by SDB with associated decreased or diminished respiratory effort. Symptoms include excessive daytime sleepiness and nocturnal awakenings (Aurora et al., 2012). Given the symptom overlap with OSA, a proportion of patients referred for the assessment of OSA will actually have an element of CSA or a mixture of the two. The WP device detects CSA by combining PAT signal changes with respiratory movements derived from its snoring and body position sensor. A large study compared simultaneous WP-200 and PSG analysis of a population undergoing assessment for SDB. The study population was enriched with individuals with congestive cardiac failure, who have a high risk for CSA due to the Cheyne–Stokes breathing pattern. These investigators demonstrated moderate sensitivity (72%), high specificity (99%), and high correlation (r > 0.8) between PSG and WP (Pillar et al., 2020) in the detection of central apneic events, suggesting yet another use of WatchPAT technology.

### Cognitive impairment and sleep disturbance

There is a strong relationship between Alzheimer's disease (AD) and sleep disturbance, and many patients with mild cognitive impairment (MCI) or AD report sleep problems. Performing PSG can be challenging in this population, due to the complexity of the equipment and also because patients are removed from their accustomed home environment. A study compared patient-reported sleep disturbance with WP sleep assessment in individuals with AD and MCI. Through this, it was found that AD patients had significantly reduced REM sleep with increased light sleep, compared to healthy controls and MCI. The authors noted that there was no correlation between subjective reports of sleep quality and objective sleep measures by WP, suggesting that WP may be a useful tool for objective sleep evaluation (Tadokoro et al., 2020).

## Conclusion

Sleep apnea and its symptoms, including sleepiness and fatigue, are common in the general population. Additionally, the COVID-19 pandemic and the fatigue associated with post-COVID syndrome may lead to an increased demand for sleep services that are already stretched by pandemic-related disruptions.

The WatchPAT is an unobtrusive device for home diagnosis of OSA. It has good correlation with polysomnography, although the results should be interpreted with caution at the extremes of disease severity. Its use has been studied and validated in a variety of patient populations, including children, older adults, pregnant women, and those with co-morbidities. The device has also been adopted, though not widely, in screening for cardiac arrhythmias and central sleep apnea.

Finally, the WatchPAT has been shown to decrease the time to OSA diagnosis and treatment and also has a cost-benefit for patients as part of a telemedicine service. It is clear that the WatchPAT could provide a cost- and time-effective solution in healthcare services where there is a high demand for services and limited access to PSG.

## Author contributions

CC and IS drafted the article, revised it critically, and approved the version to be published. All Authors contributed equally to the manuscript.
